# Pig welfare and ethical considerations during abattoir stunning: CO_2_ vs. alternative methods such as argon gas

**DOI:** 10.3389/fvets.2025.1542798

**Published:** 2025-03-13

**Authors:** Jenny L. Mace, Andrew Knight

**Affiliations:** ^1^Faculty of Health and Wellbeing, University of Winchester, Winchester, United Kingdom; ^2^School of Veterinary Medicine, College of Environmental and Life Sciences, Murdoch University, Murdoch, WA, Australia; ^3^School of Environment and Science, Griffith University, Nathan, QLD, Australia

**Keywords:** pig slaughter, stunning, CO_2_ stunning, argon stunning, inert gas stunning, pig welfare

## Introduction

Pigs are the fourth most commonly slaughtered species used for food, after fish, chickens, and ducks (1). In 2022, an estimated 1.49 billion pigs were slaughtered globally. Within the UK for example, more than 11.4 million pigs, sows, and boars (hereafter, pigs) were slaughtered in UK slaughterhouses in 2022 (2). By late 2024 this equated to nearly a million pigs monthly, or nearly a quarter of a million weekly (3). In 2023, there were 84 slaughterhouses accepting pigs in the UK, with 10 of these specializing in pigs insofar as 95% or more of the animals slaughtered were pigs (4).

Stunning aims to render pigs unconscious before being killed and processed. The vast majority (88%) of pigs in England and Wales are stunned and killed using high concentration CO_2_, with electrical stunning being used for most of the remaining 12% (5). Since 2003, there have been calls for the phasing out of high concentration CO_2_ (6, 7), which have been reiterated more recently (8).

In the following, we provide a brief review of the animal welfare concerns associated with CO_2_ stunning. These are then compared with the welfare concerns associated with alternative stunning methods. Welfare concerns arising from preslaughter handling and restraint for each method are also considered. This review does not cover religious slaughter because: 1) Judaism and Islam do not permit the consumption of pig flesh (9), and 2) religious (e.g., Shechita and Halal) slaughter doctrines normally proscribe methods that both stun and kill animals, which may occur with CO_2_ stunning, as is required when CO_2_ is used within the UK, for example (10).

## High concentration CO_2_ stunning: animal welfare concerns

As just mentioned, the use of CO_2_ to stun pigs needing also to kill them is required in the UK by *The Welfare of Animals at the Time of Killing (England) Regulations* (11). In the so-called paternoster system, groups of pigs enter a cage that descends into a 4–8 m deep CO_2_ pit. CO_2_ is heavier than air so it remains in the pit. The cage of dead pigs then ascends to the other side, whilst more pigs enter behind them. It can be imagined as a type of underground Ferris wheel; indeed, these cages are often referred to as “gondolas” [e.g., see (12)]. The other main system is the dip-lift system, which involves just one cage of a group of pigs descending and then ascending into and out of a pit in a straight line.

In both systems, there is a slight CO_2_ gradient between 70% and 90+% concentrations (8). CO_2_ is considered particularly aversive at concentrations above 30%, but pigs can detect it in concentrations as low as 15% [(13), p. 16]. Despite this, there is a limited incremental increase in CO_2_ levels; pigs in the next cage down from the entrance already face 70% CO_2_ concentrations [(7), p. 100]. This stands in contrast to the CO_2_ stunning of chickens, where CO_2_ is increased in multiple phases, beginning from 0–5%. It is not clear whether a shorter bout of higher intensity pain and distress is preferable to longer-lasting bouts of less intense pain and distress (14). In the UK, pigs must descend to the maximum CO_2_ concentration within 30 s (11).

The main animal welfare problems arising when pigs are subjected to this process are pain, fear, and respiratory distress, particularly as it takes an average of 30 s for pigs to lose consciousness (8). These welfare problems are chiefly indicated by gasping, vocalizations (squeals), and escape attempts [(8), p. 69]. These indicate aversion and negative affective states such as distress, which may be profound. These behavioral indicators are exhibited by a majority of pigs; for instance, Jongman et al. (15) found that 63% to 82% of pigs displayed gasping behavior. They also found that pigs displaying more aversive reactions took longer to lose consciousness. Whilst some convulsions such as leg kicking may occur or may continue after consciousness is lost, there is no doubt that these aforementioned behavioral indicators of poor welfare do occur whilst the pigs are still conscious (8, 16).

CO_2_ is an aversive stimulant to pigs, which is why they respond in these ways. CO_2_ irritates the mucosal lining of the trachea and nostrils, and when combined with natural bodily moisture, carbonic acid can also form on the eyes [(8), p. 65]. CO_2_ causes acidosis (acidifying blood and tissues) and hypercapnia (excessive levels of CO_2_ in the blood), which creates a sense of breathlessness, hyperventilation, and “air hunger” [(8), p. 65]. This is a significant animal welfare concern (17). Ultimately, unconsciousness is initiated by the cerebrospinal fluid of the brain becoming too acidic and the brain ceasing to function. It takes an average of 3–5 min for pigs to die within these CO_2_ pits (8).

## Alternative stunning methods: animal welfare concerns

Supplementary Table 1 summarizes alternatives to high concentration CO_2_ for stunning pigs that are discussed within the scientific literature. Based on current knowledge, the least aversive alternative to CO_2_ appears to be the use of inert gases within controlled atmospheric stunning (CAS). From purely a welfare perspective, nitrogen, argon, xenon, and helium all seem comparable in terms of their impacts on pigs; that is, whilst the welfare compromises and indicators are similar to those for CO_2_, these are generally to a much lesser degree (18). Additionally, these methods retain the ability to keep pigs in small groups and avoid more aversive handling/restraint or less reliable means of inducing unconsciousness associated with other methods. Significant handling and restraint may be required for alternative methods such as electrical or mechanical stunning. These can also cause distress to pigs (16).

When considered more broadly (penultimate column of Supplementary Table 1), it becomes clear that argon is the most promising candidate. Whilst both xenon and argon are heavier than air, making containment of the gases easier, xenon is much more expensive than argon, which is already more expensive than CO_2_. According to the European Food Safety Authority [(8), p. 70] pigs also experience a quicker time to loss of consciousness in argon (13–18 s) than in CO_2_ (17–25 s). However, there is a longer time until death with argon (roughly 7 min) than with CO_2_ (roughly 5 min). The two extra minutes required for death would impact abattoir throughput rates.

Throughput rates have risen over recent decades, during which there has been a pattern of a declining number of slaughterhouses, each with a higher throughput rate (4). The UK Agriculture and Horticulture Development Board describes some of the disadvantages of an overreliance on a lower number of large-scale slaughterhouses, including tailbacks in the chain if one plant loses its operational capacity either temporarily or longer term. Thus, alongside animal welfare reasons for reducing throughput rates (19), there are other strategic reasons too. Alternatively, there could be consideration of a two-phase CAS approach whereby argon is used to stun the pigs, followed by the subsequent addition of CO_2_ to kill the pigs [(20), p. 8]. This could allow throughput rates to be preserved.

Nevertheless, it is important to note that pigs still experience hypoxia in argon, and die from brain hypoxia, without immediate loss of consciousness [(18), p. 67]. Air hunger and gasping may still be evident, albeit to a far lesser extent (8). For these reasons, argon remains far from an ideal alternative to CO_2_. With all CAS methods, it is the actual stunning process that is the cause of negative welfare. Thus, it becomes especially difficult to argue how the practice provides a “humane death”—a key purpose of stunning. Whilst a holistic view must indeed be taken when choosing a stun method, including consideration of related stressors such as those arising from handling or restraint, this should not mean that the need for the immediate onset of unconsciousness becomes flexible. On the contrary, if no feasible stun method exists that can provide acceptable animal welfare outcomes, then from ethical and animal welfare perspectives, the species concerned should not be slaughtered in abattoirs.

Low atmospheric pressure stunning (LAPS) has recently been developed as a possible alternative. However, as indicated by a welfare score of −2 in Supplementary Table 1, LAPS provides the poorest animal welfare outcomes. With recent research from the UK Department for Environment Food and Rural Affairs and the Humane Slaughter Association (21) demonstrating severe ear pain due to barotrauma in pigs killed by LAPS, this method seems worse than the current CO_2_ method.

Of note is that the American Veterinary Medical Association (18) and Grandin (16) stated that some pig breeds react more negatively toward high concentration CO_2_ than others. On this basis, they suggested a genetic solution to the distress that many pigs experience. Additionally, efforts could be directed toward minimizing stress in earlier areas of the abattoir by disallowing the use of electric prods to hurry pigs onward, permitting pigs to move at a normal walking speed, using light to encourage pigs into new areas, using nonslip floors, enhanced training in animal welfare amongst staff, pig enrichment in holding areas, amongst many other proposals (16, 22). However, working on such genetic and logistical solutions would not change the fact that CO_2_ is aversive to pigs. These strategies may or may not reduce the severity of distress in the worst cases, but would not remove the main distressing stimulus. Efforts could instead be directed into these genetic and logistical solutions for the continued (albeit lower levels of) aversion experienced by pigs when using inert gases, rather than CO_2_.

Despite the continued welfare shortcomings of argon, the use of argon in CAS for the stunning and killing of pigs is recommended as an alternative to CO_2_ stunning. Given its shared attributes with CO_2_ (importantly, being heavier than air), current systems should be able to switch to argon use with some adjustment. To minimize costs for industry, research should investigate the possibility of recycling the gas to compensate for the slightly higher cost, as also suggested by Sindhøj et al. (20) and Jongman et al. (12).

Urgent research is still required to find a gas (or another method) that combines minimal handling/restraint, group processing, and instantaneous and reliable stunning. Switching to argon should be considered a much needed stopgap until a less aversive solution is found. Although the associated welfare impacts remain significant, and are still not acceptable from ethical or animal welfare perspectives, in the authors' opinions, they represent a significant improvement on CO_2_ stunning.

## Conclusions

This brief review summarized the welfare impacts of pig stunning and slaughter using high concentration CO_2_. It then examined alternatives to CO_2_ and compared the welfare impacts of both the actual stunning/killing procedure, as well as any handling/restraint required. Based on the research available, the use of the inert gas argon poses the fewest welfare problems for pigs, and the fewest obstacles for industry in terms of implementation. Accordingly, the industry is recommended to seamlessly switch to argon within the controlled atmospheric stunning systems already in place, whilst expediting further research into more instantaneous and non-aversive means of stunning and slaughtering pigs.
