# Supplementary material for: Pig welfare and ethical considerations during abattoir stunning: CO2 vs. alternative methods such as argon gas
Source: Front Vet Sci. 2025 Mar 13;12:1542798. doi: 10.3389/fvets.2025.1542798 (PMC11948533; doi:10.3389/fvets.2025.1542798)
Supplement: Supplementary file 1 [file Data_Sheet_1.pdf]

Table 1. Summary of the most significant welfare indicators and problems arising from the main pig slaughter methods suggested as alternatives to CO_2 gassing.

Note: For each alternative method, columns summarise requirements for handling/restraint, welfare hazards, indicators and concerns, whether loss of consciousness (LOC) is normally instantaneous, and whether the method may be used to stun without concurrent killing. Based on these factors, a score of + or - 1, 2 or 3 is provided to denote mild, moderate, or marked compromises or improvements of welfare respectively, compared to CO_2 gassing, in the assessment of the authors. Equal scores do not necessarily imply zero welfare differences between methods, but rather that any differences were not assessed as sufficient to warrant different scores. Additionally, a scale of 3 does *not* indicate ideal welfare; this would be impossible with any killing of healthy animals, as premature death in and of itself is recognised as a welfare problem due to the elimination of the potential for future positive welfare (23). The main disadvantage of each method compared to CO_2 gassing is also provided, as is a supporting citation(s). The stunning/slaughter methods conferring the *least* welfare concern (when considering both handling/restraint and stunning method) are highlighted in green.

<table>
<tr><th>Category</th><th>Stunning / slaughter method</th><th>Associated handling / restraint method</th><th>Main welfare indicators (hazards in italics)</th><th>Welfare concerns / hazards (in italics)</th><th>Instant LOC if correctly executed</th><th>Simple stun</th><th>Welfare score cf. CO_2 (-3 to 3)</th><th>Main disadvantage cf. CO_2</th><th>Citation to support</th></tr>
<tr><td rowspan="8">Contr-olled atmos-pheric stunning (gas addition)</td><td>Lower CO_2 concentra-tions</td><td rowspan="8">Minimal, and only mechanical (group)</td><td rowspan="8">Escape attempts, high pitched vocalisations, injuries, turning back, reluctance to move, gasping, intense breathing, hyperventilation (for noninert gases), headshaking</td><td rowspan="8">Pain, fear, respiratory distress</td><td>No</td><td rowspan="8">Illegal in the UK; must be stun-kill.</td><td>0</td><td>Longer time to LOC with similar welfare concerns</td><td>(8)</td></tr>
<tr><td>Inert gas: Nitrogen</td><td>No</td><td>2</td><td>Lighter than air (harder to contain)</td><td>(8)</td></tr>
<tr><td>Inert gas: Argon</td><td>No</td><td>2</td><td>Higher cost, longer time to death</td><td>(8. 15)</td></tr>
<tr><td>Inert gas: Xenon</td><td>No</td><td>2</td><td>Much higher cost</td><td>(7, 15)</td></tr>
<tr><td>Insert gas: Helium</td><td>No</td><td>2</td><td>Lighter than air, much higher cost, longer time to death</td><td>(15, 20)</td></tr>
<tr><td>Inert gas and CO_2 mix</td><td>No</td><td>0</td><td>Longer time to LOC with similar welfare concerns</td><td>(8, 20)</td></tr>
<tr><td>Nitrous oxide</td><td>No</td><td>0</td><td>Similar welfare concerns, potent greenhouse gas</td><td>(15)</td></tr>
</table>

Electrical	Electromagnetic radiation (microwave irradiation)	Loading and restraint individually	Reluctance to move, *incomplete stun / regaining consciousness: spontaneous breathing, eye reflexes, vocalisation, raising of head*	Fear, restriction of movement, *incomplete stunning, regaining conscious prior to slaughter, pain*	Yes	Yes	0	Restraint	(7, 15, 20)
	Electrical: head-only (manual or automated)				Yes	Yes	0	Restraint/handling, high chance of regaining consciousness	(8)
	Electrical: head-to-body (automated)			Fear, restriction of movement, *pain*	Yes	No	0	Restraint/handling	(8)
Mechan-ical	Penetrative captive bolt	Restraint (nose noose)	*Unsuccessful stunning: no collapse, spontaneous breathing, eye reflexes, vocalisation,*	*Fear, pain*	Yes	Mix	-1	High risk of incomplete stunning (pigs are a difficult species for this method as their brain is positioned deeper within the skull than in cattle)	(8, 18)
Miscellaneous	LAPS (low atmospheric pressure stunning)	Minimal, and only mechanical (group)	Vocalisations (grunting, high-pitched), facial grimace, escape attempts, headshaking, head tilting, possible ataxia whilst conscious	Pain, fear, disorientation, distress	No	Not yet in use	-2	Lengthy induction of LOC, pain, barotrauma	(16, 20, 21)
	Nitrogen foam	Minimal, and only mechanical (group)	Escape attempts, increased heart rate	Pain, fear, distress	No	Not yet in use	-1	Aversive and lengthy induction of LOC	(15, 24)
